# The 2016 World Humanitarian Summit Report Card: Both Failing Marks and Substantive Gains for an Increasingly Globalized Humanitarian Landscape

**DOI:** 10.1371/currents.dis.a94dd3e2f84d0a5abc179add7286851c

**Published:** 2016-08-30

**Authors:** Deon V. Canyon, Frederick M. Burkle

**Affiliations:** Daniel K. Inouye Asia Pacific Center for Security Studies, Honolulu, Hawaii, USA; Harvard Humanitarian Initiative, Harvard University, Cambridge, Massachusetts, USA; The Woodrow Wilson International Center for Scholars, Washington, DC, USA

## Abstract

Outcomes of the World Humanitarian Summit were mixed with some refreshing new directions being endorsed and a lack of systemic reform. The selective agenda and OCHAs lack of success in engaging pre-meeting political participation not only hampered the Summit’s ability to deal with global issues and institutional reform, but also alienated it from leading aid agencies and governments. The UN’s failure to commit to humanitarian principles and global disarray of the humanitarian system indicates the need for extensive reform or a new global humanitarian body. This agency needs to employ a decentralized model to manage aid funds, assume coordination of international responses, resolve civil-military coordination, cater for people affected by both conflict and disasters, and professionalize the humanitarian career.

## The 2016 World Humanitarian Summit Report Card

The World Humanitarian Summit (WHS) in Istanbul took place on May 23-24, 2016 with 9000 attendees from humanitarian aid and disaster relief organizations, crisis-affected countries and governments. It was in part prompted by the terrific flow of refugees from the Middle East to Europe, the growing gap in being able to meet the needs of displaced peoples affected by conflict and disasters and the realization that broad reform is essential to move forward.

The UN Office for the Coordination of Humanitarian Affairs (OCHA) conducted pre-WHS consultations with non-governmental organizations (NGOs), academia, youth, and the private sector on 13 October 2015. This was followed by a global meeting on 14-16 Oct to capture the opinions, ideas and voices of the multitude of stakeholders. 1 ^,^ 2

OCHA attempted to attract a large number of global leaders to the summit with all indication of conducting a formal intergovernmental process. However, when only 50 non-G7 leaders indicated attendance,^3^ representing a paltry 5.2% of the global population, it may have determined that an ‘unofficial’ exploratory approach would be more useful in advancing the reform agenda at this stage in the process. Unfortunately, nation states prefer to participate when the outcomes are clear and may have felt disinclined to attend due to a lack of extensive political involvement and dialogue.^4^ However, the tightly orchestrated program left little room for exploration and emerging issues had to be discussed in casual conversation.

Based on the outcomes of the pre-WHS process and the resulting agenda and participant list, some decided not to attend. Most notable was the absence of Médecins Sans Frontières (MSF), which was frustrated by the lack of attention to international humanitarian law and civilian protection – two issues that greatly affect its own ability to operate and function effectively in conflict-prone environments.^5^ In 2015, 150 health facilities had been bombed, and in Syria over 220 systematically targeted attacks were launched on separate medical facilities making a shambles of any “law of war” protections in the 21st Century.^6^ The inclusion of these elements was a reasonable expectation since the summit was oriented towards organizations engaged in humanitarian assistance, which needs to be bundled with legal and physical protection. Likewise, this neglect was a slight on cooperation with the security sector, which is invariably called upon for assistance as soon as governments and NGOs cannot cope. Clearly, today’s wars are pursued on the same barbaric belief last seen centuries ago: ‘the more egregiously and violent the war is waged the shorter it will be.’

Conflict results in displaced peoples crossing borders and subsequent international assistance. Much of what happens in humanitarian action is thus about politics and security, which requires the support of international law and the enforcement of accepted standards. Nevertheless, no clarity was forthcoming on the required conditions that must exist for a UN agency to initiate humanitarian action and exactly what relevance the Geneva Convention requirements have today. Without these issues of law being resolved, the protection of assisters and the assisted continues to be problematic.

The organizers made the best of the situation and abandoned the prospect of political solutions and serious reform in favor of smaller, more achievable objectives. The outcomes reflected this predicament and manifested in the form of many fairly technical commitments, such as the ‘Grand Bargain,’ the name given for a package of reforms to humanitarian funding designed to make humanitarian assistance more ‘effective and efficient.’ However, despite considerable attention to technical financial solutions, the reforms were accused of being watered down during the negotiation process. Not much emerged to fill the humanitarian aid gap or address the need for flexible multiyear financing and longer time-frames. It is hoped that efforts will now turn towards monitoring action and accountability to avoid depressing effects on the ground.

There were also very positive outcomes with promising endeavors in the areas of localization, innovation and education. A fundamental shift on how to provide humanitarian assistance, especially in situations of protracted conflict, was promoted in a new business model for the sector. The UN and international donors were asked to move from ‘What action we take’ to ‘What action you take with our support’ to foster action by local organizations. The WHS provided a platform for an international conversation by humanitarian practitioners on the need for change and its endorsement of this shift lends the process new energy and direction. Under the Emergency Medical Team movement, local and national capacity should grow in leaps and bounds once the UN and other local, national and international humanitarian players adopt this model.^7^

This shift in the way that humanitarian assistance is provided is nothing new and some organizations have been transferring the burden of local effort to local organizations for a long time while they move command and control further afield. Other trends in innovation, recognizing the role of education and the relief-development relationship in protracted conflict and urban situations were inspiring.^8^

It is argued that the formal humanitarian system “faces a crisis of legitimacy, capacity and means, blocked by significant and enduring flaws that prevent it from being effective”.^9^ This is not only due to increased frequency, magnitude, severity, duration and complexity of crises. It is due to the UN failing to follow its own principles and breaking its own system.

Unfortunately, following the WHS, a scathing report revealed that the UN, by abdicating control of the delivery of aid in Syria to the Assad government, is in serious breach of the humanitarian principles of impartiality, independence and neutrality directly resulting in the prevention of aid to those most in need.^10^ In the 1980s in Africa, subsequent Security Council resolutions sanctioned government interference in the delivery of food and medical aid. This resulted in unmitigated civilian deaths and starvation, but the UN has repeated this breach of principle. This represents a dramatic departure from the first goal of the WHS to “Reaffirm our commitment to humanity and humanitarian principles”^11^ and the second core responsibility – to “uphold the norms that safeguard humanity.”^2^

The WHS was in a way, recognition that the humanitarian system is dysfunctional. It encouraged the participation of a large number of people to foster open and productive dialogue in a massive information-sharing endeavor. It facilitated explicit and elevated, sector-wide, formal and informal policy discussion on humanitarian systems, structures and funding.^8^ However, it failed to adequately address systemic issues and focused on piecemeal technical solutions.

Humanitarian aid is no longer only about the UN and donors. We need to move away from the current highly centralized model in which local and national NGOs only receive 0.2% of OECD Development Assistance Committee aid funding while UN agencies receive the bulk.^9^ A naturally evolving ecosystem of diverse actors that collectively represent and work for common humanitarian objectives is arising. The need to identify how to work cooperatively is driving the call for reform, which is the next logical step that will influence how civil society, political entities and the security sector work together in the years to come.

The WHS was successful in promoting the idea of local ownership and leadership in humanitarian action, and this is an essential precursor to a structural reform of the humanitarian system. The summit has created pressure for change that is expected from the next, soon to be elected UN Secretary General. Subsequent reform meetings should focus on why the current humanitarian system exists, who it serves, what behaviors it encourages, how this impacts on the delivery of aid, how the world has changed, and how humanitarian standards and laws need to evolve to continue to be relevant and functional. While powerful political support is required, it is the donors that hold the purse strings and it is them that need to support this reform.

We need a global humanitarian body employing a decentralized operational model to manage the flow of funds from governments and the cloud, and coordinate international responses. Such an agency would be able to end the divide between aid organizations and the security sector and would cater not only for people affected by conflict, but also disasters. Such an agency would be able to step in when small nations are unable to coordinate responses to overwhelming crises. The over 210,000 people working in humanitarian assistance also need legitimization which can be achieved by professionalizing the humanitarian occupation.^12^^,^ 13 The UN has demonstrated a failure to commit to its own humanitarian principles and is no longer qualified to lead this effort without far more significant reform.
